# Inquiries Concerning the Intellectual Powers and the Investigation of Truth

**Published:** 1831-04-01

**Authors:** 


### Medico-Chirurgical Review,

No. XXVIII.

JANUARY 1, to APRIL 1, 1831.

### I.

Inquiries concerning the Intellectual Powers and the Investigation of Truth. By *John Abercrombie*, M. D. &c. Octavo, pp. 435. Waugh and Innes. London, 1830.

It has been usual to consider the thinking principle as a very compound being; consisting of parts perfectly distinct, and executing functions perfectly different. It is certainly true that we can perceive the qualities of external objects—that we can compare these qualities with each other—that we can decide upon their resemblances, or dissimilarities—that we can associate these judgments into groups—combine them into connected trains of reasoning—recall, at pleasure, ideas and combinations of ideas formed long before, and fancy perceptions and associations, which never had any object. These are mental operations, which are hourly performed, and of which we feel undoubtingly conscious. But it appears more than questionable, whether these few facts entitle a division of the thinking principle into distinct parts and separate properties.

The essence of mind we know nothing of, the exercise of mind we cannot explain, and its operations are frequently as mysterious as its nature. When a material object is brought within the sphere of our senses, by examining it attentively we become acquainted with its colour, consistence, form, and size. We are said to perceive these qualities, and the knowledge, which is thus acquired, is said to consist of simple perceptions. But, after obtaining these perceptions of external objects by this exercise of the senses, the mind proceeds to operate upon these materials in various ways. She reduces them into various forms—she devotes them to various purposes—she recalls before her each perception individually—abstracting her attention from every other perception, she directs herself exclusively to the examination of one—she decides upon, or forms a judgment of its nature—she compares the judgment which she has formed of one perception with the judgment to which she has arrived respecting another, and, by reasoning on the qualities of both in connexion, she forms an opinion, not merely of their abstract, but of their relative properties—she then classifies these perceptions according to their qualities, and estimates them according to their resemblances—she generalizes upon the qualities of objects as yet unseen, by her knowledge of the perceptions of those which she conceives to be either similar, or opposite—and, last of all, she can imagine a thousand qualities and combinations of matter, which have no existence, and of which she has therefore never had any knowledge, but which, from her extensive knowledge of the qualities and combinations of matter which do exist, and which she has perceived, she can invest with similar but modified properties. Thus, then, do metaphysicians say, is it evident that we have perception, attention, memory, recollection, reflection, abstraction, judgment, reason, conception, causation, imagination, and many other mental functions.

Still, however, there is strong reason for believing, that these are not so many distinct and different functions, or properties of mind, but the varied and modified workings of an indivisible, unique and unmembered principle; and that any difference, which may appear between them, depends more upon the degree in which mind is exercised in each, than upon any essential difference in their original constitution. When we view a portrait, we cannot confine the exercise of mind, which sight of such an object calls forth, to a mere act of perception. We cannot avoid forming some opinion of the features of the figure, of their size, proportion, or symmetry. If the nose be large, or the eye small, or the colours vivid, or the hair grey, we cannot refrain from considering them as such the moment they are seen. Our simple perception of the object and this opinion of it appear to be almost, if not altogether, simultaneously formed; and, although by more minute examination we may criticise the piece with more exactness, and evince much greater depth of mind in our remarks, still it is only by more minute perception that more delicate judgments are formed, and to the taste of an artist, which education has developed and experience has refined, the detection of a blemish, or the discovery of a beauty is only to be seen to be decided on.

Although general authority is unfavourable to this view, yet we have some most distinguished metaphysicians who strongly favour it. Dugald Stewart has done much to simplify the science of mind; Dr. Browne has done more, and Mr. Mill, in his late admirable "Analysis of the Human Mind,"* has cheered us with the hope, that the period is not distant, when the subtleties of Descartes and Reid shall be consigned to the same oblivion with the ancient categories and absurd distinctions of Aristotle.

For the sake of perspicuity and more convenient expression, do we believe, has mind been chopped down by many writers into such fractional divisions; but, in consequence of our strong bias to convert mere names after lengthened use into actual entities, this metaphysical dissection has introduced more difficulty and disorder into the study of mind, than can be easily imagined by any, save those who have been doomed to encounter them. Mason Good's Nosology is superior, both in euphony and intelligibility, to some catalogues of mental functions which we have seen; and, although perhaps they scarcely equalled it in the number, they certainly were not inferior to it in the minuteness of their subdivisions. If any advantage can be gained by attributing to mind distinct functions, and by distinguishing these functions by specific names, the arrangement of all the mental faculties into perception, memory, judgment, and imagination is the best. Sensation is only a form of perception, attention is merely the direction of perception to a specific object, abstraction is a specific appli-

* See the Westminster Review for October, 1830.

cation of attention, conception is a renewed or recollected perception, recollection is a form of memory or memory in exercise, causation is a function of judgment, and reflection is a conjoint exercise of judgment and memory. Unless, therefore, we admit the doctrine of intuitive ideas, which is a metaphysical paradox unworthy of the great men by whom it has been advocated, all our knowledge is derived from the exercise of these four mental powers, or rather from these four varieties of mental exercise—perception, memory, judgment and imagination; and the confused and confounding jargon of the schools, which describes the human mind as infinitely more complicated than the most mysterious piece of mechanism which ever ingenuity invented, should be exposed by every friend to plain and useful knowledge.

But, while we maintain the simplicity of mind, and contend that metaphysics is indebted for the repulsive aspect, which it presents to many minds, to our unlogical and unmeaning mode of studying it, we wish it to be understood that we are only referring to the operations, or effects of the thinking principle, not to the thinking principle itself. The thinking principle is not an object of study in the abstract, because in the abstract we can know no more of it, than we do of the independent, invisible spirit, from which it emanates and by which it exists. We are acquainted with a few of its properties, we know a few of the principles according to which it works, we can arrange some of its operations, and we can invest, with an imposing garb of scientific nomenclature, this meagre alphabetical metaphysics. But we are all this time drudging among the outworks; we are learned only in signs and in symptoms. The thinking principle is as deeply immersed as ever in inscrutible profundity, and the imperfect information which we possess on the whole subject of mind (excluding its simple operations) amounts to little more than a scholastic classification of abstruse terms, and a profound description of superficial phenomena. Matter is known to us only by its properties, and a few even of these properties are sufficiently understood; and is it to be conceived that matter, which is subject to every sense, which comes into contact with our every mean of knowledge, which is palpable to the touch, visible to the eye, and accessible by every avenue of thought, should still remain unknown and as mysterious as ever, and yet that mind, which is immediately cognizable by no sense but that of consciousness, which can be seen only through its works, and even then as through a glass darkly, and whose absence can be predicated only by finding no traces of its operation—is it to be conceived that this being is to be denied an independent life, because we cannot subject it to the rule and plummet, bring it within the field of our microscope, weigh it in our scales, or measure it by our yard-stick! What we call mind has, probably, no connexion with what we call matter in any other district of the universe, than on the present globe—at least there is nothing known contrary to such a supposition—and we do know that in other parts of the universe, what we call mind exists and exercises its functions apart from and independent of matter. The present connexion of matter with mind is, therefore, as far as we know, the only instance in which they are united, and, exclusive of every other proof, this is one reason for believing that the operation of mind and mind itself are just as independent of matter as matter can possibly be of mind. When it is said, then, that mind is immaterial in construction, incomprehensible in essence, independent of matter in its existence, and in being after the matter, with which it has been for a time in connexion, is dissolved, we only assert what analogy teaches, what the distinctive peculiarites of mind and matter warrant, what scientific observation infers, and what revelation confirms. "Of all the truths we know," says Mr. Stewart, "the existence of mind is the most certain. Even the system of Berkely, concerning the non-existence of matter, is far more conceivable than that nothing but matter exists in the universe." We have the evidence of consciousness for the existence of the one, but only that of sense for the being of the other; and if the thousand derangements, to which our external senses are obnoxious, be considered, the assurance which we possess of the existence of mind is much less equivocal, and may be more surely depended on, than any which mere sense can furnish to prove that there is any other creature than mind in the universe.

The stumbling-block of most materialists consists in the intimate connexion which subsists between mind and matter in our present state of being. They cannot, or they will not see that this connexion, although intimate, is unessential. The very intimacy of this connexion is confounded with identity of being, with oneness of substance, of property and of life. Because spiritualists admit that the brain is the organ, through which mind manifests itself, materialists insist that mind is a secretion of the brain. Because those allow that soundness of brain is necessary for sanity of mind, and that disease of the former is followed by disorder of the latter, these argue that mind cannot operate without brain, that organization is indispensible to thought! This is a sad shipwreck of all reasoning—a melancholy abandonment of legitimate induction. Dr. Abercrombie's remarks on this metaphysical monstrosity are excellent.

"The brain, it is true, is the centre of that influence on which depend sensation and motion. There is a remarkable connexion between this organ and the manifestations of mind; and by various diseases of the brain these manifestations are often modified, impaired, or suspended. We shall afterwards see that these results are very far from being uniform; but even if they were uniform, the facts would warrant no other conclusion than that the brain is the organ of communication between the mind and the external world. When the materialist advances a single step beyond this, he plunges at once into conclusions which are entirely gratuitous and unwarranted. We rest nothing more upon this argument, than that these conclusions are unwarranted; but we might go farther than this, and contend, that the presumption is clearly on the other side, when we consider the broad and obvious distinction which exists between the peculiar phenomena of mind, and those functions which are exercised through the means of bodily organization. They do not admit of being brought into comparison, and have nothing in common. The most exquisite of our bodily senses are entirely dependent for their exercise upon impressions from external things. We see not without the presence both of light and a body reflecting it; and if we could suppose light to be annihilated, though the eye were to retain its perfect condition, sight would be extinguished. But mind owns no such dependence on external things, except in the origin of its knowledge in regard to them. When this knowledge has once been acquired, it is retained and recalled at pleasure; and mind exercises its various functions without any dependence upon impressions from the external world. That which has long ceased to exist is still distinctly before it; or is recalled, after having been long forgotten, in a manner even still more wonderful; and scenes, deeds, or beings, which never existed, are called up in long and harmonious succession, invested with all the characters of truth, and all the vividness of present existence. The mind remembers, conceives, combines, and reasons; it loves, and fears, and hopes, in the total absence of any impression from without, that can influence, in the smallest degree, these emotions; and we have the fullest conviction that it would continue to exercise the same functions in undiminished activity, though all material things were at once annihilated.

This argument, indeed, may be considered as only negative, but this is all that the subject admits of. For when we endeavour to speculate directly on the essence of mind, we are immediately lost in perplexity, in consequence of our total ignorance of the subject, and the use of terms borrowed from analogies with material things. Hence the unsatisfactory nature of every physiological or metaphysical argument respecting the essence of mind, arising entirely from the attempt to reason the subject in a manner of which it is not susceptible. It admits not of any ordinary process of logic, for the facts on which it rests are the objects of consciousness only; and the argument must consist in an appeal to the consciousness of every man, that he feels a power within totally distinct from any function of the body. What other conception than this can he form of that power by which he recalls the past, and provides for the future; by which he ranges uncontrolled from world to world, and from system to system; surveys the works of all-creating power, and rises to the contemplation of the eternal cause? To what function of matter shall he liken that principle, by which he loves and fears, and joys and sorrows; by which he is elevated with hope, excited by enthusiasm, or sunk in the horrors of despair? These changes also he feels, in many instances, to be equally independent of impressions from without, and of the condition of his bodily frame. In the most peaceful state of every corporeal function, passion, remorse, or anguish, may rage within; and, while the body is racked by the most frightful diseases, the mind may repose in tranquillity and hope. He is taught by physiology, that every part of his body is in a constant state of change, and that, within a certain period, every particle of it is renewed. But, amid these changes, he feels that the being whom he calls himself remains essentially the same. In particular, his remembrance of the occurrences of his early days, he feels to be totally inconsistent with the idea of an impression made upon a material organ, except he has recourse to the absurdity of supposing that one series of particles, as they departed, transferred the picture to those which came to occupy their room." 34.

"We know nothing of the nature or the essence of mind; but whatever may be its essence, and whatever may be the nature and extent of that mysterious connexion which the Deity has established between it and our bodily organization, these points have no reference whatever to the great question of its future existence. This momentous truth rests on a species of evidence altogether different, which addresses itself to the moral constitution of man. It is fouud in those principles of his nature by which he feels upon his spirit the awe of a God, and looks forward to the future with anxiety or with hope;—by which he knows to distinguish truth from falsehood, and evil from good, and has forced upon him the conviction that he is a moral and responsible being. This is the power of conscience, that monitor within, which raises its voice in the breast of every man, a witness for his Creator. He who resigns himself to its guidance, and he who repels its warnings, are both compelled to acknowledge its power; and, whether the good man rejoices in the prospect of immortality, or the victim of remorse withers beneath an influence unseen by human eye, and shrinks from the anticipation of a reckoning to come, each has forced upon him a conviction, such as argument never gave, that the being which is essentially himself is distinct from any function of the body, and will survive in undiminished vigour when the body shall have fallen into decay." 37.

"There is thus in the consciousness of every man a deep impression of continued existence. The casuist may reason against it, till he bewilder himself in his own sophistries; but a voice within gives the lie to his vain speculations, and pleads with authority for a life which is to come. The sincere and humble inquirer cherishes the impression, while he seeks for farther light on a subject so momentous; and he thus receives with absolute conviction the truth which beams upon him from the revelation of God,—that the myterious part of his being which thinks, and wills, and reasons, shall indeed survive the wreck of its mortal tenement, and is destined for immortality." 38.

Whether we regard mind as divisible into parts, or indivisibly entire, its first exercise, as has been already stated, in acquiring knowledge of the external world, is perceptive, and the first fruits of that exercise are simple ideas. But as these ideas are obtained through the senses, any derangement in the corporeal function of sensation must produce a corresponding aberration in the mental faculty. Dr. Falconer relates the case of a man, whose sensation was so morbid, that cold bodies felt to him as though they were hot. A patient mentioned by Baron Larrey, on recovering from amaurosis, saw men as giants and all objects greatly magnified. We are acquainted with a gentleman who, on recovering from fever, detected the taste of colchicum in every wine which was offered to him ; and the author gives us the particulars of a patient, who, while sitting alone in his drawing-room during twilight, the door of his room being half open—"saw distinctly a female figure, wrapped in a mantle, and the face concealed by a large black bonnet. She seemed to advance a few steps towards him, and then stop." He was always, however, able to convince himself of the illusion, by observing that he could see through the figure, so as to distinguish the lock of the door and other objects which lay behind it. Sir Walter Scott's late work on Demonology and Witchcraft gives many very interesting instances of a similar kind.

But, although all the senses may be perfectly healthy, they often require assistance and correction from each other, in order to convey faithful ideas of external objects to the mind ; and the success, with which one sense can be made to perform the duties of another by frequent and careful application, is strikingly exemplified by many curious anecdotes. Mr. Saunderson, the blind mathematician, could by his sense of touch outstrip the most experienced virtuoso, in detecting counterfeit from genuine Roman medals. Dr. Moyse, the celebrated blind philosopher, could distinguish a black from any other coloured coat, by the peculiarity of its smell. The cases of two blind men are given by the author, who were such excellent judges of horses, that they could detect defects which had escaped the notice of more gifted, but less acute observers. One of these, in giving his opinion of a horse, declared him to be blind from the peculiarly cautious manner in which he put his feet to the ground; and the other pronounced a horse blind of one eye, by perceiving that it was colder than the other. We have heard it positively asserted of a blind man, that he could tell the color of any cow in a large flock, if he heard her roar ; and a story, little short of this in eccentricity, has been lately published, in which a gentleman, who had lost every sense but that of touch in one cheek, kept up communication with his family, by tracing characters on the part which retained its sensation.

"Much ingenuity has been bestowed upon attempts to explain, how, with two eyes, we see only one object ; and why that object is seen erect, when we know that the image on the retina is inverted. All that need be said upon the subject, and all that can properly be said, appears to be, that such is the constitution of our nervous system. It is on the same principle, that by the sense of touch, in which may be concerned a thousand or ten thousand distinct points of contact, we receive the impression of only one body ; or, what perhaps may appear a more strictly analogous case, we receive the impression of but one body, though we grasp the substance with two hands, or with ten distinct fingers. For the healthy perception in both these cases, however, a certain arrangement is required, which we may call the natural harmony of the nervous system ; and, when this harmony is disturbed, the result is remarkably altered. Thus, squinting produces the vision of a double image, because the images fall upon what we may call unharmonizing points of the retina; and the same principle may be illustrated, in a very curious manner, by a simple experiment with the sense of touch. If a small round body, such as a pea, be laid upon the palm of the one hand, and rolled about between the first and second fingers of the other, in their natural position, one pea only is felt; but, if the fingers are crossed, so that the pea is rolled between the opposite surfaces of the two fingers, a most distinct impression of two peas is conveyed." 55.

Some have attempted to account for this singular phenomenon in vision on the principle of habit, forgetting that habits are only formed in the course of time and after much attention, and that when formed they merely consist of a facility of action, acquired by its frequent repetition. Others have gone so far as to say that inverted objects are at first seen as they are represented, and that experience ultimately corrects what was at first an error. Descartes accounts for it by saying, that the notice, which the mind takes of the object seen, depends not on the image painted upon the retina, but on the situation of that portion of brain from which the filaments of the optic nerve arise. Mr. Molyneux makes the whole matter very easy by asserting, that it is not the eye which sees, but the soul, and that it is to investigate the essence of a subject, which we know nothing of, to enquire how the soul sees inverted images erect; and Mr. C. Bell treats the subject quite as lightly, since, according to him, the terms above and below have no relation to the image in the bottom of the eye, but to the position of our bodies and surrounding objects.

We have collected these different views on this difficult question, for the sake of shewing how easily some points of a most interesting nature are passed over, even by celebrated writers, and how strongly we are inclined to make any reply to a question pass for its solution, rather than honestly acknowledge we know nothing at all about it. None of the ingenious authors, whom we have mentioned, could have been himself satisfied with his own solution, and however we may cover and conceal the difficulty in such unintelligible jargon, as soul-seeing and brain-seeing, it must be evident to any candid inquirer that there not only is a difficulty in the subject, but that this difficulty still remains unexplained.

How with two eyes we see objects singly is also a matter of considerable obscurity. Descartes, who was never backward in grappling with ultimate facts, and reducing them to reason, supposed that the nervous filaments, which compose the retina in both eyes, are homologous, that each filament has a corresponding part of the brain in each hemisphere, and that when any impression is made upon these filaments, the corresponding portions of brain only are affected at the same instant. Sir Isaac Newton ascribed single vision with double images to decussation of the optic nerves behind the sphenoid bone, adopting the opinion of Galen and the old anatomists. Gassendi and M. Du Tour thought that the mind attended to one image only at once; Dr. Reid refers the explanation to an original conformation of the eye and mind; and others have maintained that originally all objects making two pictures—one in each eye—is imagined to be double, but that by degrees we find, when corresponding parts of both retinæ are impressed, both images represent but one object. Mr. Cheselden gives the case of a gentleman, who had one of his eyes distorted from a blow inflicted on the head, and who, for a long time after the accident, saw all objects double. By experience, however, this deception was gradually rectified, the most familiar objects at first began to appear single, and, although the obliquity of the injured organ still continued, every thing was ultimately seen without presenting a duplicate aspect.

The knowledge, which we obtain through the external senses, is extremely limited. Were one confined to this single source of information, our attainments in knowledge could never rise to any distinguished degree of excellence. But, by storing up the ideas which have been obtained through the senses, by combining, comparing, classing and deducing inferences from them, a new fund of thought is established, which is not much inferior to the former in productiveness, and at advanced life is the principal and often the only fountain from which knowledge is acquired. Metaphysicians have, therefore, divided our ideas into such as are procured from sensation and those which are derived from reflection—into simple and complex ideas, ideas original and secondary. The first comprehend all such as are communicated to the mind through the medium of our senses, whether they be immediately perceived by personal observation, or mediately received through the testimony of others. The second comprehend all ideas that arise from memory, reflection, abstraction, consciousness, reason, imagination and other exercises of mind, which have less immediate direction to external objects than to their images, which sensation has already furnished. The number, nature and even the existence of the latter class of ideas depend upon the perfection and variety of the former. The one class form the foundation, the other the superstructure of all human knowledge.

The Doctor arranges, under the three following heads, all the ideas which we derive from reflection :—

"I. A knowledge of the mental processes, and the laws and relations by which they are regulated ;—a knowledge, for example, of the laws and facts relating to memory, conception, imagination, and judgment. These will be more particularly referred to in a subsequent part of our inquiry. In the same manner we acquire our knowledge of those which have been called the active and moral powers, as love, hope, fear, joy, gratitude, &c.

II. Certain notions arising out of the exercise of the mental processes, in reference to the successions and relations of things ;—our notion, for example, of time, arising out of memory and consciousness ;—our notion of cause,—of motion,—number—duration—extension or space. From simple perception we seem to acquire a knowledge of external things as existing only at the moment ; and from simple consciousness a knowledge of a mental impression as existing only at the moment.—Our notions of the succession of things, as implying time and motion, require the exercise of consciousness and memory ; and our notions of cause, and the various other relations of things to each other, require both memory and comparison. To the same head, in reference to another department of these faculties, belong our notions of truth and falsehood,—right and wrong. These result from a certain exercise of mind, aided by that remarkable principle in our constitution, which commonly receives the name of conscience.

III. With this exercise of the mental functions, there spring up in the mind certain convictions, or intuitive and instinctive principles of belief. They are the immediate result of a certain exercise of the understanding, but are not referable to any process of induction or chain of reasoning, and can be considered only as an original and fundamental part of our constitution. This is a subject of great and extensive importance, and the articles of belief which are referable to it, are chiefly the following :—

(1.) A conviction of our own existence as sentient and thinking beings, and of mind as something distinct from the functions of the body.

(2.) A confidence in the evidence of our senses in regard to the existence and properties of external things ; or a conviction that they have a real existence independent of our sensations.

(3.) A confidence in our own mental processes ;—that facts, for example, which are suggested to us by our memory, really occurred.

(4.) A belief in our personal identity, derived from the combined operation of consciousness and memory ; or a remembrance of past mental feelings, and a comparison of them with present mental feelings, as belonging to the same sentient being.

(5.) A conviction that every event must have a cause, and a cause adequate to the effect.

(6.) A confidence in the uniformity of the operations of nature ; or that the same cause, acting in the same manner, will always be followed by the same effect." 70.

We apprehend that the Doctor has arranged within his third head some ideas, which cannot be considered as original and fundamental parts of our constitution, but are strictly referrible to a chain of reasoning, however fine or imperceptible. For instance, how do we gain confidence in the uniformity of nature's operations? Fundamental parts of our constitution, or more properly speaking instincts, should be as complete in childhood as at a more advanced age, yet the infant mind, which has no experience of the laws of nature, and no knowledge of the properties of matter, can be certain of neither, and cannot safely predicate any thing of either. It is a law of nature that a stone, when thrown into the air, will always return to the surface of the earth, or that hydrogen, when formed into air-bubbles, will leave the earth and rise into the air. But how does the boy, who amuses himself with these pastimes, come to the knowledge of these facts? Not, assuredly, by intuition, nor instinct, nor any fundamental principle of his constitution ; but by a close and attentive process of reasoning. He has either seen a stone, when thrown into the air, fall back again to the earth, or he has been told by those, whom he has reason to believe, that when so circumstanced it will do so. He then tries it himself, it succeeds as he anticipated, and he is then convinced by repeated trials that as often as he repeats the experiment the same result will succeed. All this knowledge is the fruit, not of instinctive impulse, but of reasoning, however elementary and simple it may appear. So much so is it the fruit of reasoning and experiment, that were he not convinced by a similar experience, that his air-bubbles would rise without being projected and never fall again, his mere knowledge of the stone's tendency to return to the earth when removed from it, would unquestionably induce him to predicate the same result from his experiment upon the air-bubble. The fact is that it is our very experience, our POST FACTA knowledge of the laws of nature which makes us so confident of their regularity, and many facts could be advanced to prove that the experience, which we possess of their regularity, is the precise and only measure of our confidence. Were an ignorant peasant to see, for the first time, a steam-boat, at some distance upon the ocean, plying at the rate of ten miles an hour in direct opposition to a strong tide and a boisterous wind, without sails or oars, or any discoverable mode of propulsion ; or were he to see, under the same circumstances, the æronaut seated in a ponderous car, ascending from the earth like some huge inhabitant of the air, and sailing along the heavens with the rapidity of an eagle, all that he ever before knew of the laws of Nature, all that he had before seen, was obviously opposed to these laws in these phenomena, and the mysterious agency of some superhuman being would probably be considered necessary to reconcile all that he witnessed with the palpable evidence of his own senses. Yet, let a more enlightened observer, whose experience is more expanded, whose acquaintance with the thousand combinations into which matter can be wrought is more minute, and who knows how her inmost and most essential laws can, as it were, be employed as the most effectual agents against each other—let such an observer be brought for the first time to witness the motion of the steamer against wind and water, and the ascent of the car against the force of gravity, and, although as ignorant of the machinery employed in either case as the unlettered and bewildered peasant, and as unable as he to form the least conjecture as to the *cur* or the *quomodo* of what is passing, still he will rest satisfied that all is done in both cases in strict accordance with the laws of Nature, and that his present perplexity and ignorance are no objections against principles, which he has ever found as universal in their existence, as they have been faithful in their operation. When the King of Siam was informed by a Dutch traveller, that at certain seasons of the year in Holland water became so solid, that an elephant could walk with firmness upon its surface, he replied—"I have believed many extraordinary things which you have told me, because I took you for a man of truth and veracity, but now I am convinced that you lie." The King of Siam was a true epicurean; he admitted no evidence but that of sense. He had never seen solid water, and the chances of water becoming solid under any circumstances appeared to his mind so very few and feeble, that he considered it more prudent to discredit the testimony which asserted such an event, than attach faith to a circumstance, so different from every thing he had himself witnessed.

To object to every evidence, but that of sense, merely because the thing attested is opposed to our personal experience, is weak in the extreme; yet, strange to say, this is the very essence of Mr. Hume's celebrated argument against the practicability of miracles under any circumstances. As every one admits, argues this sophistical and dangerous moralist, that the evidence of sense is superior to the evidence of testimony, as my senses have invariably borne witness to the uniformity of Nature's laws, and as not only none of my senses have ever witnessed the performance of a miracle, but the performance of a miracle supposes the violation of these laws which my senses inform me are never violated, I run less hazard in wholly rejecting as false the evidence of testimony, it matters not how strong, which attests the existence of a miracle, than were I to believe as true what is diametrically contradicted by my evidence of sense. Now, admitting this reasoning to be just, it follows that we are to credit nothing which is not in perfect harmony with our own personal, immediate knowledge, and, therefore, when the steam-boat was first seen sailing against the wind, or when the æronant was first seen ascending in his car, both phenomena should have been considered as physical impossibilities by all, who had not witnessed their occurrence with their own eyes. According to Hume, no amount of human testimony could undeniably attest appearances so contrary to all that had been before seen, so opposed to all that he was pleased to call "unalterable experience;" and on the same ground we are advised to reject, without even examination, miracles, however generally and strongly attested, as so many cheats upon public faith.

How a man, who was one of the first historians of his day, could pride himself in the acuteness and impregnability of such wretched jesuistry, can only be explained by those, who are experimentally acquainted with the hollow emptiness of the Deist's system. If nothing contrary to our own unalterable experience can be attested by any weight of evidence, how did he give credit to much of what he himself wrote and published and expected others to believe? Were it necessary, after Dr. Campbell's unanswerable reply to Hume's Essay on Miracles, we could point out passages and narrations in his History of England, which are received by him and retailed to the world as true, which, notwithstanding, are more difficult of belief, because more opposed to our experience, than most of the miracles which he argues no amount of evidence could attest. If we can discover inconsistency or absurdity in the substance of another's testimony, then we are called upon to discredit it, not merely because it is inconsistent with our own experience, but more especially because it is inconsistent with itself—not because the event narrated has never been seen, or heard of by any other but the narrator, but because it is in its very nature contradictory and unreasonable. Its external evidence may be suspicious enough, but its internal evidence should alone condemn it.

The grand object of Hume, Tindal, and every other deistical writer, has been to throw ridicule upon even the possibility of a miracle, by endeavouring to show that it is an absurdity, and therefore no longer an object of knowledge;—but

"This leads us to a very obvious distinction of extraordinary events,—into those which are only marvellous, and those which are to be considered miraculous. A marvellous event is one which differs in all its elements from any thing that we previously knew, without being opposed to any known principle. But a miraculous event implies much more than this, being directly opposed to what every man knows to be the established and uniform course of nature. It is farther required that such an event shall be of so obvious and palpable a kind, that every man is qualified to judge of its miraculous character, or is convinced it could not happen from the operation of any ordinary natural cause.

In receiving a statement respecting such an event, we require the highest species of testimony, or that, on which we rely with the same confidence as on the uniformity of the course of nature itself. But even with this amount of testimony, a doubt may still remain. For we have two amounts of probability which are equally balanced against each other; namely, the probability that such testimony should not deceive us, and the probability that there should be no deviation from the course of nature. The concurring evidence of numerous credible witnesses, indeed, gives a decided preponderance to the testimony; and upon a certain amount of testimony we might receive any statement, however improbable, as in the case admitted by Mr. Hume, of a universal darkness. But, though in such a case we might receive the statement as a fact which we could not dispute, the mind would be left in a state of absolute suspense and uncertainty in regard to any judgment which we could form respecting it. Something more appears to be necessary for fixing the distinct belief of a miraculous interposition; and this is an impression of moral probability. This consists of two parts. (1.) A distinct reference of the event to a power which we feel to be capable of producing it, namely—a direct interposition of the Deity. (2.) The perception of an adequate object, or a conviction of high moral probabillty, that an interposition of Divine power might be exerted in such circumstances, or for the accomplishment of such an object. Such are the miracles of the sacred writings. As events opposed to the common course of nature, they are, by the supposition, physically improbable in the highest degree. Were they not so—were they in the lowest degree probable according to our conceptions of the course of nature, they could not be miracles, and consequently could not answer the purpose for which they are intended. But notwithstanding this species of improbability, they carry with them all the elements of absolute credibility—namely, the highest species of testimony, supported by a moral probability which bears directly upon every element of the statement." 83.

There is nothing absurd in supposing that water has been converted into wine, although we ourselves may never have witnessed such conversion; whereas it were very absurd to suppose that, after the water had been changed into wine, it still continued to possess and present its own natural and distinctive properties. Had it at one time been asserted that water could be changed into air, and that air could be converted into light, the narration would have been deemed marvellous, probably as marvellous as it appeared to the King of Siam that water could be solidified, or to Hume that it could be changed into wine; yet these are both natural phenomena, and, although we mean not to insinuate, that the conversion of water into wine depended on any law of Nature, applicable at least to the present economy of things, yet we hold that all these four conversions must appear equally singular to an inexperienced mind, and there would be equally good reason for discrediting the possibility of either on the ground, that nothing should be believed which was opposed to personal experience.

It is very little short of nonsense when any man talks of his unalterable experience. However extensive his opportunities for observation, however expanded his talents, however wide and comprehensive his acquirements, his store of facts must be very scanty, and his experience of to-day will be altered by the occurrences of to-morrow. As observation multiplies experience increases; new inferences are found, old opinions are modified, and deductions, which had been once considered just, are discovered to be inaccurate. Our experience, consequently, even of the laws of Nature, is changing hourly, unless we cease to observe the changes which occur hourly around us, and events, which would at one period of the world have been esteemed impossible, or would have been ascribed to the operation of some unearthly influence, can now be traced with unerring certainty to some principle, which had been then equally in operation, but was utterly unknown. Our author has made many excellent observations in harmony with the spirit of these strictures, but space will not permit us to extract them. In his sketch of the intellectual operations the following passage occurs on the influence of arbitrary association, which we cannot deny ourselves the pleasure of bringing forward.

"It is unnecessary to enlarge upon the subject of arbitrary association, as the observation of every one will furnish numerous examples of it. There is one application of the principle, however, which deserves to be referred to in a more particular manner. I allude to the practice of commemorative rites, or periodical observances, for transmitting the remembrance of remarkable events. These are, in their nature, in general, entirely arbitrary; or, if they have any analogy to the events, the relation is only figurative. But the influence of such celebrations is of the most extensive and most important kind. If the events, particularly, are of a very uncommon character, these rites remove any feeling of uncertainty which attaches to traditional testimony, when it has been transmitted through a long period of time, and consequently, through a great number of individuals. They carry us back, in one unbroken series, to the period of the events themselves, and to the individuals who were witnesses of them.

The most important application of the principle, in the manner now referred to, is in the observances of religion which are intended to commemorate those events which are connected with the revelation of the Christian faith. The importance of this mode of transmission has not been sufficiently attended to by those, who have urged the insufficiency of human testimony, to establish the truth of events which are at variance with the common course of nature. We have formerly alluded to one part of this sophism, and have stated the grounds on which we contend, that no objection to the credibility of these events can be founded upon our observation of what we call the course of nature. We have admitted, that a much higher species of evidence is required for them than would be required for events which correspond with our previous observation; and this high and peculiar evidence is confirmed in a striking manner by the periodical rites now referred to. By means of these we are freed entirely from every impression of the fallibility of testimony, and the possibility of the statements having been fabricated; as we are conducted in one uninterrupted series, to the period when the events took place, and to the individuals who witnessed them. This will appear, if we state in a few words a hypothetical case. Let us conceive a person attempting to impose upon the world, by an account of some wonderful or miraculous event, which, he alleges, occurred 500 years ago. He, of course, exerts every possible ingenuity in fabricating documents, and framing the appearance of a chain of testimony in support of his statement. It is quite possible that he might thus deceive a considerable number of credulous persons; and that others, who did not believe his statement, might yet find difficulty in proving its fallacy. But, if the report were farther to bear, that, ever since the occurrence of the alleged event, it had been regularly and specially celebrated by a certain periodical observance, it is clear that this would bring the statement to the test of a fact open to examination, and that the fallacy of the whole would be instantly detected.

On these principles it must appear, that the statements of the sacred writings, respecting miraculous events which are said to have occurred upwards of 1800 years ago, could not have been fabricated at any intermediate era during that period. It is unnecessary to state, how much more improbable it is, that they could have been fabricated at the very time and place in which they are said to have occurred, and in the midst of thousands who are said to have witnessed them, many of whom were deeply interested in detecting their fallacy. This part of the question is not connected with our present inquiry, but it is impossible to dismiss the subject without one reflection:—that if we are to proceed upon the principle of probabilities, we must balance fairly the probabilities of fabrication. If we do so, we hesitate not to assert, that the probability of the world being imposed upon, under all the circumstances now alluded to, is more at variance with our firm and unalterable experience, than all that we are called upon to believe." 125.

The Dr.'s account of the action and progress of disease upon the mind is extremely interesting, in tracing it up from the first aberration of thought to confirmed insanity; and as we have devoted as much of our space to what may be more strictly considered the metaphysics of this volume, as can well be expected in a medical review, the author must not conclude by our omitting to notice several sections, that they are inferior to the context in execution.

Attention is supposed to be the first mental function which bodily disease impairs; memory is soon affected by consequence, for although our perceptions may be perfectly correct, it is attention alone which renders their recollection sure; and as memory deteriorates, every other mental exercise becomes impaired. Objects being first imperfectly perceived and afterwards indistinctly recollected, the judgment is misled by misrepresented qualities, and minconceived resemblances. A man mentioned by Mr. Abernethy, who was a native of France, but had spent the greater part of his life in England, received an injury on the head, after which his French was perfectly restored to him, although previously to the accident he had quite lost the habit of speaking it. Dr. Prichard cites the case of a lady, who, during a fit of delirium, addressed her attendants in a tongue which no one at first understood, but which was afterwards discovered to be Welch. For a long time it was matter of mystery to her friends, how she became acquainted with a language so very different from her own; but it was discovered after minute inquiry, that during infancy she had been nursed by a woman, who had formerly belonged to the coast of Brittany. A gentleman, whose case is given by Dr. Beattie, received a blow on his head, and with it immediately vanished every recollection of the Greek language, with which he had before been sufficiently conversant.

Nothing can more strikingly illustrate the very intimate influence, which corporeal disease exerts upon purely mental operations, weakening, deranging, and even wholly disabling them, than such accidents as we have now recorded, and the materialist has not failed to turn this circumstance to his account. But, if it be fair to ground upon a morbid state of the system an argument, which is to apply to a natural condition, it cannot be refused us to turn the weapons of our opponents against themselves, and to show how frequently the body is woefully, irrecoverably diseased, without materially affecting the mental faculties. How often do we find the shadowy, shivering skeleton, whose material system has been wasted by protracted illness into a frightful atomy, with a perception as acute, a memory as faithful, a judgment as correct, and an imagination as chaste, as though the patient were in the enjoyment of the most brilliant state of health. Disease may have brought existence to the very verge of extinction, and the dilapidated tenement may be heaving its last expiration upon the margin of the tomb, and yet the soul may be as elastic in its powers, and as energetic in their exercise, as when this frail and temporary frame-work stood strong in all the vigour of unimpaired manhood. The skull has been shattered, and large portions of the brain have been mashed into shapeless pulp, and yet mind has maintained undisturbed possession of her throne, unshook by the violence thus committed in her neighbourhood. Dr. Abercrombie, in his work upon the Brain, has narrated the case of a lady, in whom one half of her brain was reduced to a diseased mass, yet who retained all her mental powers to the last, and had formed one of a convivial party a few hours before death. A man is described by Dr. Ferriar, who died suddenly, but in the full exercise of mind, although upon inspection it was found, that the right hemisphere of his brain was completely suppurated; and Mr. O'Halloran mentions a man, who sustained such an injury of the head, that a large portion of the scull was removed on the right side, and through this opening a large quantity of matter, mixed with portions of brain, was discharged at each dressing,. This went on for seventeen days, until nearly one half of the brain was destroyed; yet through the whole of this affection the mind steadily maintained its vigour, and even at the moment of dissolution betrayed no sympathy with such frightful disorganization. How such facts as these can be met by the materialists, they are perhaps best prepared to say; but one thing is certain, that there is no organ in the whole body so sensible to injury as the brain, which could perfectly elaborate its secretions under such disease, or unexceptionably perform its functions under such disadvantages. Such facts the Dr. very truly observes—

"Show us indeed, in a very striking manner, the mind holding intercourse with the external world, through the medium of the brain and nervous system; and, by certain diseases of these organs, they show this intercourse impaired or suspended; but they show nothing more. In particular, they warrant nothing in any degree analogous to those partial deductions which form the basis of materialism. On the contrary, they show us the brain injured and diseased to an extraordinary extent, without the mental functions being affected in any sensible degree. They show us, farther, the manifestations of mind obscured for a time, and yet reviving in all their original vigour, almost in the very moment of dissolution. Finally, they exhibit to us the mind, cut off from all intercourse with the external world, recalling its old impressions, even of things long forgotten; and exercising its powers on those which had long ceased to exist, in a manner totally irreconcileable with any idea we can form of a material function." 156.

On the other hand, how often do we see the most intemperate insanity—an utter wreck of all the mental functions—without being able to detect the slightest structural derangement? The works of Esquirol, Pinel, Haslam and Burrows, bear undeniable attestation to this fact, and every practitioner of any consequence must often meet with proofs of it within the range of his own experience. The brain shall appear quite sound—neither too soft nor too hard, neither too pale nor too vascular, neither diminished nor enlarged, neither externally malformed by Nature nor internally altered by disease;—yet the perception shall be false, the memory shall be faithless, the judgment shall be deranged, the imagination shall be extravagant, and the whole mind shall be disqualified from performing one series of connected thought. It is certainly no proof that the body is not somewhere, or somehow diseased, because we can discover none;—but it is a proof that derangement of mind bears no proportion to derangement of matter, and that no argument in favour of the materiality of mind can be drawn from pathology.

Next to that of dissecting the thinking principle into a thousand members, no mistake appears to us more productive of confusion upon this subject, than that of regarding insanity as a distinct and specific malady, composed of ingredients so exclusively its own, that they can never exist where it is absent. As it is generally understood it is considered a peculiar and almost hopeless derangement of the thinking principle. The insane are regarded as labouring under a strange and anomalous bereavement; as severed from society by some signal dispensation of misfortune, which rightfully excludes them from the privileges, and almost from the sympathies of humanity. The leprosy was never more esteemed among the Jews as a judicial infliction, than madness is with us. The leper and the maniac are equally avoided, equally outcast, equally treated with a repulsive feeling of aversion; and the day has not long gone by, when mental derangement under every form was ascribed to the presence of some demoniacal influence. It is a fact, however, as indisputable as it is important, that the idiot, the lunatic and the madman are nearly allied to the man of prudence, the man of talent, and the man of high and varied erudition. Eccentricity is seen every day, and therefore it passes without observation; but eccentricity is only one form of derangement. The difference between eccentricity and mono-maniasm is merely a difference of degree, and any other difference, than in extent, between monomaniasm and confirmed madness it were difficult to specify. *Nemo mortalium omnibus horis sapit* loses nothing of its force by being an old and hackneyed sentiment; and could we persuade the world that mental derangement, in some form or degree, is universal, the outcast and deserted lunatic would stand a better chance of recovery, or of receiving better treatment should he not recover. He, who is not certain how soon he may himself occupy a cell in some asylum, will probably feel more interest in the comfort and restoration of those, who had been once rational; and if no purer principle than selfishness can be excited in behalf of lunatics, few generally can be lectured with more effect.

Mind is seldom found in a state of unexceptionable integrity, and the degrees of derangement which it suffers are so infinite, that in the largest receptacle of lunatics, which we have ever entered, no two of its inmates could be discovered who were in all respects similarly crazed.

"The peculiar character of insanity, in all its modifications, appears to be, that a certain impression has fixed itself upon the mind, in such a manner as to exclude all others; or to exclude them from that influence which they ought to have on the mind in its estimate of the relations of things. This impression may be entirely visionary and unfounded; or it may be in itself true, but distorted in the applications which the unsound mind makes of it, and the consequences which are deduced from it. Thus, a man of wealth fancies himself a beggar, and in danger of dying of hunger. Another takes up the same impression, who has, in fact, sustained some considerable loss. In the one, the impression is entirely visionary, like that which might occur in a dream. In the other, it is a real and true impression, carried to consequences which it does not warrant.

There is great variety in the degree to which the mind is influenced by the erroneous impression. In some cases, it is such as entirely excludes all others, even those immediately arising from the evidence of the senses, as in the state of perfect mania formerly referred to. In many others, though in a less degree than this, it is such as to change the whole character. The particular manner, in which this more immediately appears, will depend of course upon the nature of the erroneous impression. A person, formerly most correct in his conduct and habits, may become obscene and blasphemous; accustomed occupations become odious to him; the nearest and most beloved friends become objects of his aversion and abhorrence. Much interesting matter of observation often arises out of these peculiarities; and it is no less interesting to observe, during convalescence, the gradual return to former habits and attachments. A young lady, mentioned by Dr. Spurzheim, who had been for some time confined in a lunatic asylum, had shown, for several weeks, every mark of a sound mind except one,—she hated her father. At length, she one day acknowledged, with pleasure, the return of her filial attachment, and was soon after discharged, entirely recovered. Even when the erroneous impression is confined to a single subject, it is remarkable how it absorbs the attention, to the exclusion of other feelings of a most intense and powerful kind. I knew a person of wealth, who had fallen into a temporary state of melancholic hallucination, in connexion with a transaction in business which he regretted having made, but of which the real effect was of a trifling nature. While in this situation, the most severe distress occurred in his family, by the death of one of them under painful circumstances, without his being affected by it in the slightest degree.

The uniformity of the impressions of maniacs is indeed so remarkable, that it has been proposed by Pinel, as a test for distinguishing real from feigned insanity. He has seen melancholics confined in the Bicetre, for twelve, fifteen, twenty, and even thirty years; and, through the whole of that period, their hallucination has been limited to one subject. Others, after a course of years, have changed from one hallucination to another. A man, mentioned by him, was for eight years constantly haunted with the idea of being poisoned; he then changed his hallucination, became sovereign of the world and extremely happy, and thus continued for four years." 311.

All the principal theories of insanity we apprehend, are the offspring of one common error. One function, or department of mind has generally been fixed upon as the faculty diseased, and the selection made has too often depended upon some peculiar crotchet in the brain of the theorist, resulting from the observation of a few peculiar cases which happen to fall within his own notice. But insanity must be studied with more extent of view, and can only be thoroughly understood in its ten thousand modifications of aspect, by being seen and studied in them all. The imagination may be extravagant, the perception may be deceptive, the judgment may be diseased, the association may be disordered, or the memory may be deranged. Any one of these states of mind, when present to excess, will often so unhinge every other mental power, and so peculiarize every mental operation, as to impart to the whole conduct an air of imbecility, or actual derangement. It is impossible to trace every instance of insanity to an anormal condition of any one function, or specific exercise of mind; and it is to this attempt we are indebted for many of the crude and inconsistent speculations of writers upon this disease. The author refers to the five following heads the principal forms of mental hallucination;—1st. to propensities of character, which circumstances had restrained during health;—2dly, to old associations, recalled to the mind and mixed up with more recent occurrences;—3dly, to visions of the imagination, which had formerly been indulged in;—4thly, to corporeal feelings, and, lastly, to a vague and ill-defined consciousness, on the part of the insane, of the deranged state of his mental powers.

"Attempts have been made to refer insanity to disease of bodily organs, but hitherto without success. In some instances, we are able to trace a connexion of this kind; but, in a large proportion, we can trace no bodily disease. On this subject, as well as various other points connected with the phenomena of insanity, extensive and careful observation will be required, before we are entitled to advance to any conclusions. In regard to what have been called the moral causes of insanity, also, I suspect there has been a good deal of fallacy, arising from considering as a moral cause, what was really a part of the disease. Thus, we find so many cases of insanity referred to erroneous views of religion, so many to love, so many to ambition, &c. But, perhaps it may be doubted whether that which was in these cases considered as the cause, was not rather, in many instances, a part of the hallucination. And even when the mind does give way under a great moral cause, such as overwhelming misfortunes, we often find that the hallucination does not refer to them, but to something entirely distinct;—striking examples of this are mentioned by Pinel." 325.

There is reason to believe that we are unable to fathom the Doctor's real meaning in this passage, because, were it not for other expressions in his book which discountenance the doctrine, the language here employed would incline us to suspect, that he considered mind itself capable of disease. This view we are convinced Dr. Abercrombie does not advocate, and therefore we must ascribe any suspicion, which may appear in the extract now given, to the inaptitude of the terms in which it is written, rather than to the sentiments which these terms were intended to convey. Bodily disorder of some kind or other is, unquestionably, the sole cause of insanity of mind. It is impossible to attach, even in conception, disease to any thing purely spiritual, and, although the corporeal derangement may escape observation, and only manifest its existence by unhinging the understanding, that is no ground for inferring, that it is the understanding itself which is primarily diseased.

Many curious observations are made on the subjects of spectral illusions, somnambulism and dreams, but we are compelled to pass on to the fourth part of the Doctor's volume, which is, at least in a medical point of view, more important than either of the three which have been so hastily run over. Indeed, we feel somewhat disappointed that a physician of such experience and talent—to whom the deficiencies of our present mode of *medical instruction* must appear glaringly obvious, and the weight of whose authority upon such a subject could not fail to call the public attention to its pernicious effects—should have appropriated so small a portion of his space to the application of philosophy to medical science. We should not, however, feel surprised if the Doctor have only reserved himself for a future opportunity;—if so, we shall be among the foremost to stamp with our official passport any thing, which may emanate upon this subject from such a quarter;—anticipating as we can from the sample now before us, that the honor which it would confer upon the writer would be trifling when estimated with the advantage which would accrue from it to the profession. Mind may be imperfectly known in other departments of science, and ignorance in the elements of reasoning may mar the general progress of knowledge; but—sorry are we to say it—in no profession are such defects so extensively prevalent, or so mischievously effective as in medicine. Metaphysics are scarcely ever thought of as an *essential* component of even a physician's education, and, although in Oxford, Cambridge and Dublin, the preliminary studies of a medical graduate's course *nominally* embrace the science of mind, if we are to follow an old, but sensible rule of judging of its benefits—by their fruits you shall know them—we shall find but little reason for making these seminaries of learning exceptions to our general criticism. When the hurried curriculum of study, which a general practitioner is required to run through, is considered—when we know that twelve months' lectures, at almost any hedge-school of medicine, entitle him to an examination before the chartered authorities—when we examine the nature and the extent of the acquirements which are sufficient to pass these grave tribunals with glowing honors—and when we know how much requires to be read and seen and studied, to qualify the most gifted mind, surrounded and aided by the most favourable circumstances, for treating disease in its ten thousand forms—we can easily see, why such a thing as mind and pure science are wholly overlooked in this hastened preparation, and why the whole attention is bent upon the more palpable and payable preparatives for practice. It is an infinitely more pressing study to know the colors, and the smells, and the weights, and the prices of drugs—to know the art of labelling bottles, of directing cards, of gilding pills, and of administering lavements—to acquire the tactics of professional manœuvering, of getting introduced to influential circles, of undermining the progress of rival practitioners, and of pleasing the senses of our patients by arranging the colors of our mixtures and the tastes of our draughts. These are the *first elements of medical science*, and, as upon the first rudiments in every branch of knowledge depend future success and more general attainments, it is methodical enough to settle the foundation ere we commence the superstructure. Æsculapius prevent us from saying ought disrespectfully of the art and mystery of medicine, or of needlessly intermeddling with the shades and savors of its drugs! It may be prudent to bribe as many of the senses of our patients as we can, that they may report favourably of our pills and potions; and when one is under the necessity of being physicked, he may as well be purged or vomited by medicines wearing his favourite colors;—this may all be little affairs of *trade*, which it were only vexatious and meddlesome to say much concerning; but the sin which we are raising our voice against is the habit, the universal custom of devoting so much attention, of ascribing so much importance, and of wasting so much time on learning these *rudiments of quackery*, while the very grammar of the science lies still unopened and very probably despised.

The uncertainty of medicine and the obscurity of disease are no reasons why disease should not be treated with all the facilities, which a competent education can afford, and why medicine should not be studied with all the care and enlightenment, which any other scientific subject of pursuit demands. The very difficulty of an investigation is the strongest reason, why the mind of the investigator should be more than commonly prepared for it; and he who chuses a profession, because many of its principles are unsettled, and because ignorant presumption may often be mistaken for enlightened confidence, is only a gambler in speculation, who wishes to cover his own incompetency under the hazards of the game. There is no doubt but such speculation occasionally secures its object, and that fame and fortune are the stakes it wins; but it is equally certain, that it more frequently miscarries, and although we cannot hope that any argument, founded upon its immorality, can have much effect with such minds as engage in it, we are not acquainted with any form of gambling half so immoral in its principles, or half so ruinous in its effects.

The science of medicine is, certainly, not like the science of mathematics, nor the science of numbers, nor like any of the exact sciences. Its principles are not in every instance reducible to demonstration, and its details too often flow without the precision of direct and necessary sequences; notwithstanding, medicine is a pure science. Many of its principles are as precise and as fixed, and many of its details can be as safely inferred and as certainly depended on, as those of any of the more exact sciences; and we maintain that much, if not most of the opprobrious uncertainty, which is so vehemently railed against by some, and humourously boasted of by others, is the unmixed result of the incompetency, indifference, and venality of its professors. The public have lately taken greater liberties with the medical practitioner than they ever before attempted. Charges of ignorance and indictments for mal-practice would, some years ago, have been regarded as monstrous, if not miraculous:—they were never heard of—they were never thought of; but *tempora*, and I believe we may add, *cum hominibus mutantur*. The medical practitioner cannot at present deliver a woman, or open a vein, or adjust a fracture, or perform the very most trifling operation, without having the terrors of a judgment-seat constantly before his eyes; and if the dread of law can effect, what a love of science and a respect for the interests of humanity should have secured, we fear it will do but little in restoring him to public confidence.

In studying any subject, the first thing to be done is to collect as many facts relating to it as we have reason to believe are sufficiently accurate;—in the second place, these facts should be carefully compared with each other, and arranged according to their resemblances, or dissimilarities;—in the third place, we should trace any connexion which may exist between these facts, so as to ascertain the nature and extent of their influence upon each other; and lastly, from these ascertained facts and arranged relations such general inferences should be drawn, as may guide us in our further investigations. These four simple rules comprehend, in the Doctor's opinion, all that is necessary to be observed in studying medicine as a science; and we have no doubt that were some such rules as these strictly attended to, in studying the laws of animal life, the nature of disease, and the action of remedies, medicine would ere long be divested of much of that empiricism, which now disgraces her, and her professors would exchange the rank and respectability of trading grocers, for that of lovers of truth and enlightened friends of humanity. In collecting our facts the following sources of fallacy should be kept constantly in view, that we may not be guilty of—

"(1.) Receiving as facts, statements which are not facts but opinions.—A person dies after being affected with a certain set of symptoms, and we find, on examination after death, the usual appearances of hydrocephalus. Another is seized with similar symptoms, and recovers. He is therefore said to have recovered from hydrocephalus, and such a statement is often given as a medical fact. The man's recovery from certain symptoms is a fact; that he recovered from hydrocephalus is not a fact but an opinion.

(2.) Receiving as a fact, a statement which only assumes the relation of facts.—A person recovers from a particular disease, while he was using a particular remedy. His recovery is ascribed to the effect of the remedy; and the cure of the disease by this remedy is often given as a medical fact. The man's recovery is a fact; and that he used the remedy is another fact; but the connexion of the remedy with his recovery we are not entitled to assume as a fact. It is tracing between the facts the relation of cause and effect,—a process of the utmost delicacy, and not to be admitted on any occasion, but with the greatest caution.

(3.) Receiving as facts, general statements, or the generalization of facts. One of the most common examples of this error occurs, when a statement is given of a symptom or set of symptoms, as certainly diagnostic of any particular disease, or of a particular morbid condition of an internal organ. Such a statement we hold to be of no value, except we have absolute confidence in the narrator, both in regard to his habits as a philosophical observer, and to the extent of the observations on which his statement is founded. But, with every possible advantage in these respects, we are to exercise the utmost caution before we receive the relation, thus stated, as a fact; for it is to be kept in mind, that it is not properly a fact, but a generalization of facts. Some writers, for instance, have maintained with much confidence, that a particular state of rigidity of some of the limbs is distinctly characteristic of ramollissement of the brain. But further observation has shown that the disease may exist without this symptom, and that this condition of the limbs may appear in connexion with other diseases. Their observation of facts was in so far correct, that this state of limbs does very often accompany ramollissement of the brain; the error consisted in giving it as a general fact, or a fact applicable to all cases of ramollissement—which is without foundation. Yet such statements, when brought forward with confidence, are often received as facts, and rested upon as established principles; and then the facts by which their fallacy might be detected are apt to be overlooked or forgotten.

This may perhaps be considered as one of the most prevailing errors in the modern science of medicine; and it is indeed astonishing to observe the confidence with which such statements are brought forward, and the facility with which they are received as equivalent to facts, without attention to the manifold sources of fallacy with which they are encumbered. Does a writer, for example, tell us he has ascertained that the spinal cord is diseased in all cases of Tetanus. If we knew that such a statement had been founded on the careful observation of an hundred cases, it would be of value; if it was deduced from a few, its value is greatly diminished. But even if it had been deduced from the larger number, certain doubts would still arise in considering the relation thus stated as a fact. We should naturally ask ourselves,—was the narrator qualified to judge of the facts and their relations,—were the cases referred to, all really cases of Tetanus,—were the appearances in the cord such as could properly be considered as indicating disease,—or might any of them have been mere changes of colour, or other incidental appearances, which might have taken place after death, or might have been the effect of the convulsion rather than its cause,—or were they such changes as may be found in other cases without any symptoms of Tetanus? Other sources of fallacy will come into view, if the statement be, that the narrator has uniformly found a certain remedy of great efficacy in a particular disease. Here, in the first place, similar questions occur as in the former instance;—on how many cases did he found his statement,—how did he ascertain the disease,—and was he qualified to decide that it really was a case of the disease which he alleges. But, supposing all these questions to be answered in a satisfactory manner, others still arise, namely,—had the alleged treatment really any influence on the recovery of the patients,—did they get well in consequence of the treatment, or in spite of it, or altogether independently of it,—have not similar cases recovered spontaneously, or under modes of treatment entirely different.—Such is the uncertainty of causation and generalization in medicine; and such is the danger of receiving general statements as equivalent to facts." 382.

In arranging these facts for classification we should separate the fortuitous from the uniform, and the suspicious from those which have been thoroughly ascertained. And here we cannot refrain from expressing ourselves in the strongest manner on a subject which the Dr. has very properly introduced —we mean an unreasonable attachment to nosological distinctions. "The nosologist," he justly remarks, "proceeds upon the principle, that the characters of disease are to a certain extent fixed and determined, like the botanical characters of a plant, or the chemical properties of a mineral." And what are the consequences?—Why, that the young and inexperienced practitioner, who goes out into the world with his memory loaded with the definitions of a favorite nosology, lays his master's rule and line to every thing which he sees and reads, and if any discrepancy from the standard can be detected the case is considered anomalous, and how much soever it may in other respects resemble a well-defined and obvious disease, the young nosologist stands confounded, and unable to proceed one step upon ground, which the limits of his lesson were not intended to embrace. One and the same disease often wears a hundred different appearances in a hundred different constitutions, and a definition of it, which would include all the symptoms which constitute these modifications, would often be so general and vague, that it would be more applicable to all than distinctive of any. The definitions, which are most useful in medicine, are descriptions; and the less the student cramps his observation under the direction of such meagre guides, the more speedily will he become acquainted with the general principles of disease. Exclusive of the fact, that the nosologist is very frequently wrong in his definition, he is often so inaccurate in his language, that he, who implicitly follows his definitions at the bed-side of sickness, will not seldom fall into dangerous mistakes. Thus, hydrocephalus implies the existence of water in the head, yet we may have water in the head without hydrocephalic symptoms, and hydrocephalic symptoms without water;—

"On this subject I shall only add the following anecdote, which I lately received from a medical man of very high intelligence. At an early period of his career as a naval surgeon, he was left in charge of a ship on the West India station, when several sailors presented themselves, with an affection of the legs, the nature of which was entirely new to him. Having expressed his difficulty to one of the officers, not medical, he was promptly told that the disease was scurvy, and that, if he examined the gums of his patients, he would find sufficient evidence. To this he replied, that the thing was impossible, because, in the nosology of Dr. Cullen, it was expressly specified, that scurvy occurs 'in regione frigida.' He was, however, soon convinced that the disease was really scurvy, though it occurred in the West Indies; and, as he added, received a most important lesson, to observe for himself, instead of trusting to systems." 387.

In tracing the connexion between different facts, so as to ascertain their relation, before we can safely infer that one is the other's cause or consequence, we should be certain that their apparent connexion is real, that it is immediate, and that it is invariable. A medicine of some reputation is given at the crisis of a disease; next day the patient is better, and on the day following all danger has passed. This improvement is very naturally attributed to the administration of this remedy, and when a case of a similar description recurs it is again employed with confidence of relief, but is found to produce no effect. Ulcerated intestines are a frequent attendant, or consequence of fever; they have, therefore, been esteemed by some as the cause of every other symptom, and that if the mucous action, which occasions them, be overcome, the fever will be cured. Diseased liver is frequently co-existent with hydrocephalus, therefore, in place of considering it a concomitant effect with the hydrocephalic symptoms, it has been ranked by some among its causes.

" A gentleman, immersed in the business or the pleasures of a great city, becomes disordered in his health, dyspeptic and hypochondriacal. He receives much good advice from his medical friend, which he professes to follow with implicit confidence, and proceeds to do so amid the anxieties of business, bad air, late hours, luxurious dinners, and nearly the total want of bodily exercise. Deriving no benefit from all that is done for him, he hears of some celebrated water, which has acquired great reputation in the cure of stomach complaints, and at length makes up his mind to resort thither, though with little hope of deriving benefit from any thing. He now lays aside all business, lives by rule, keeps early hours, and is all day long in the open air. He soon recovers excellent health, and cordially concurs in spreading the fame of the water by which a cure so wonderful has been accomplished. An anecdote has been related of a physician in London having advised a dyspeptic patient, who had baffled all his remedies, to go down and consult a celebrated physician in Inverness, whose name he gave him. On arriving there, he soon discovered that there was no such person to be found. He then returned to London, somewhat nettled at the trick which had been practised upon him, though he was obliged to acknowledge that he was cured of his disorder. On this subject we are especially to keep in mind, the extensive class of diseases which are acted upon, in a most powerful manner, by causes entirely mental. These are the numerous and ever varying maladies which are included under the terms, dyspeptic, hypochondriacal, and nervous. Many of them have their origin in mental emotions which elude observation; and a very large proportion are entirely referable to indolence, and inaction,—to that vacuity of mind attending the unfortunate condition in which there is no object in life but to find amusement for the passing hour. When, on patients of this description, the dexterous empiric produced results which the scientific physician had failed to accomplish, we are too apt, to accuse him, in sweeping terms, of practising upon their credulity. He, in fact, employs a class of remedies of the most powerful kind, to which the other perhaps attaches too little importance, namely, mental excitement and mental occupation, —the stimulus of having something to hope and something to do." 396.

In generalizing, or deducing general principles from individual facts, the grand error, into which we are disposed to fall, is drawing conclusions intended for universal application from a few personal observations, and a very limited experience. The physician of a few patients dogmatizes and dictates with as much pertinacity, as though he were the sole medical adviser of an entire colony; and whatever statements oppose his local knowledge, by whomsoever they may be made, or however they may be arrived at, they are rejected on the single ground of this very opposition. It is truly ludicrous to see some of these men with one idea, stigmatizing the experience of accumulated centuries as a bundle of unconcocted rubbish, and encountering the conclusions of extensive and enlightened observation, as hasty inferences and mistaken theory. Every village bone-setter has his facts and his principles, his secret views and his specific remedies; and, although the sphere of practice, from which these have been obtained, may not measure a mile square, the facts and principles of continents and colleges are frequently opposed to them in vain.

Every principle is worse than useless, unless it be universal; and unless a principle, designed for universal application, be well established, its universality will prove one of its most pernicious consequences. The humoralists ascribed a thousand symptoms to states of the fluids which they never proved to have a positive existence. Their lentor, viscidity, alkalescence, acidity, putrescence, dissolution were properties utterly hypothetical, although made to explain less equivocal phenomena, and it may be safely added that our doctrines of spasm, sympathy, irritation, and many other popular *principia* of the present day, are as ill established, and as loosely defined. The following observations cannot be too highly estimated.

"The benefit which a physician derives from his own opportunities of observation, in common language called his experience, is not in proportion to the period of time over which it has extended, or the number of facts which have passed under his view. It must depend on the attention with which he has observed these facts, and traced their relations to each other,—on the anxiety with which he has separated incidental relations from those which are uniform,—and the caution with which he has ventured on assuming the relation of cause and effect, or has advanced to general principles. It must depend, farther, on the jealousy and suspicion with which he has received even his own conclusions, and the care with which he has corrected them from time to time by farther observations. Finally, it must depend on the judgment with which he applies the knowledge thus acquired, to the investigation and treatment of new cases,—by tracing promptly the points of affinity between the case under his view, and those cases on which his knowledge was founded;—by discovering real points of resemblance where there is an apparent difference, and real points of difference where there is an apparent resemblance. The farther a physician advances in this course of rigid inquiry, he becomes more sensible of the difficulties with which his science is encumbered, more suspicious of all general conclusions, and more anxious to bring them to the test of minute and extensive observation;—in particular he learns to exercise more and more caution in considering any one event in medicine as the cause of another. In real acquisition, consequently, his progress is slow: for much of his improvement consists in detecting the fallacy of systems which he once considered as established, and the instability of principles in which he once confided as infallible. But these discoveries prepare the way for his actual progress, and the conclusions at which he does arrive then fall upon his mind with all the authority of truth." 420.

We must now close this volume, and we do so with feelings of respect for its author, which his former works, however valuable, were not so well calculated to inspire. The subjects handled on the present occasion are—many of them so extra-professional, and some of them so abstruse—that we did not expect a laboured treatise on the structure and science of mind from a pen, which had been long and deeply engaged in investigations of so different a character. It is true, that there is little originality attempted in these *Inquiries*, and that much of the matter which they contain we have been long made acquainted with by writers of another school; but metaphysics is, perhaps, the last field, which any man ambitious of originality would enter in quest of fruit, and our profession stand much less in need of brilliant discoveries on the essence, or exercise of the mental powers, than of a general and practical acquaintance with mind, as it is already known. This more useful information is contained in these inquiries, and the strongest argument, by which we can urge them upon the attention of our readers, will be found in the voluminous review, with which we have considered it our duty to introduce them to their notice. To conclude in the phraseology of their subject, they exhibit much clearness of perception, much precision of idea, much accuracy of association, much good taste and strong judgment, and, although last certainly not least in these days, a moral system so religiously disciplined, as can confer upon a head well-informed all the advantages of a heart well-regulated. The style is occasionally loose, and the language is not perhaps sufficiently precise for metaphysical disquisition; but, when the pursuits and avocations of the writer are considered, it is less remarkable that such slight deficiencies are found, than that they should so rarely occur.

